# Decreased neural activity and neural connectivity while performing a set-shifting task after inhibiting repetitive transcranial magnetic stimulation on the left dorsal prefrontal cortex

**DOI:** 10.1186/s12868-015-0181-3

**Published:** 2015-07-22

**Authors:** Niels J H M Gerrits, Odile A van den Heuvel, Ysbrand D van der Werf

**Affiliations:** Department of Anatomy and Neurosciences, VU University Medical Center (VUmc), Van der Boechorststraat 7, 1081 BT Amsterdam, The Netherlands; Department of Psychiatry, VUmc, Amsterdam, The Netherlands; Netherlands Institute for Neuroscience, An Institute of the Royal Netherlands Academy of Arts and Sciences, Amsterdam, The Netherlands; Neuroscience Campus Amsterdam (NCA), Amsterdam, The Netherlands

**Keywords:** Key-words, Set-shifting, Low-frequency repetitive transcranial magnetic stimulation, Functional magnetic resonance imaging, Prefrontal cortex, Connectivity

## Abstract

**Background:**

Sub-optimal functioning of the dorsal prefrontal cortex (PFC) is associated with executive dysfunction, such as set-shifting deficits, in neurological and psychiatric disorders. We tested this hypothesis by investigating the effect of low-frequency ‘inhibiting’ off-line repetitive transcranial magnetic stimulation (rTMS) on the left dorsal prefrontal cortex on behavioural performance, neural activity, and network connectivity during the performance of a set-shifting paradigm in healthy elderly (mean age 50+).

**Results:**

Behaviorally, we found a group-by-session interaction for errors on set-shift trials, although post hoc tests did not yield significant findings. In addition, the verum group, when compared with the sham group, displayed reduced task-related activity in the left temporal gyrus, and reduced task-related connectivity of the left PFC with the left postcentral gyrus and posterior insula.

**Conclusion:**

These results show that low-frequency off-line rTMS on the left dorsal PFC resulted in reduced task-related activity and network connectivity, which was accompanied by a subtle behavioural effect, thereby further corroborating the importance of an optimally functioning PFC in set-shifting.

## Background

The dorsal fronto-striatal circuit plays an important role in executive functions [1]. One of these functions is set-shifting, which refers to the ability to reconfigure task sets in a flexible manner in order to meet changing demands [2]. Patients with psychiatric (e.g. obsessive–compulsive disorder [3], schizophrenia [4]) or neurological (e.g. Parkinson’s disease [5, 6]) disorders, often suffer from executive dysfunction, presumably because of impaired fronto-striatal function.

Applying low-frequency (1–4 Hz) repetitive transcranial magnetic stimulation (rTMS) [7] over the motor cortex leads to decreased cortical excitability [8, 9], which is, depending on the intensity and duration of the stimulation, detectable up to 30–60 min afterwards [7]. It is assumed that rTMS induces similar effects in more associative areas [10, 11]. This temporary inhibitory characteristic of low-frequency rTMS can be used to simulate decreased functioning of prefrontal regions.

Such an approach can be employed in healthy participants to induce a “virtual lesion” [12] similar to that of patients with psychiatric and neurological disorders. Especially in combination with neuroimaging modalities such as functional magnetic resonance imaging (fMRI), TMS has the potential to extend our knowledge of neural circuits that are involved in psychiatric or neurological disorders and provide us with the opportunity to make causal statements about the function of certain brain areas [13]. For example, a previous fMRI study by our group showed that low-frequency off-line rTMS on the left dorsolateral prefrontal cortex (DLPFC) in young healthy participants resulted in decreased task-related activations in the frontal and visuospatial regions, and a decrease in behavioural performance, while performing a planning task [14]. Although it is increasingly recognized that alterations in brain activity often represent alterations in brain networks and functional connectivity [15], that can be induced by rTMS [16, 17], our previous analyses did not investigate whether rTMS also induced changes in functional connectivity. Since it is theorized that functional connectivity results from a synchronous neuronal firing pattern [18–20], and because inhibitory rTMS perturbs normal brain functioning, we expect that the stimulation will lead to a desynchronization in firing frequency, thus inducing a decrease in task-related functional connectivity of the stimulated area with other task-related areas (within the fronto-striatal and fronto-parietal circuits).

To further investigate the involvement of the prefrontal cortex (PFC), and connected areas, in set-shifting, a group of forty healthy (aged 50+) participants first performed a newly developed set-shifting paradigm in an MRI scanner during a baseline condition. This new paradigm mirrors the classic Wisconsin Card Sorting Task [21] with respect to switching after negative feedback, but depends less on other cognitive constructs (e.g. working memory, matching-to-sample, set-formation [22]) that are often present in set-shifting paradigms. They were then randomly assigned to receive either rTMS at the PFC (verum) or vertex (sham) during a second session, while using the first MRI scan to determine the most optimal stimulation location. We hypothesized that the verum rTMS group, when compared with the sham group, would display an increase in errors on set-shift trials, decreased activation in task-related brain areas, i.e. the dorsal fronto-striatal and fronto-parietal areas, and decreased connectivity between the left dorsal PFC and other task-related brain areas during the second session, when compared with the first.

## Results

### Demographics and characteristics

The sham and verum group were well matched with respect to age (*p* = .61), gender (*p* = .62), handedness (*p* = .60), and MMSE score (*p* = .36), but the sham group had a higher education level (*p* = .04) and estimated IQ score (*p* = .01). The groups also did not differ in BDI (*p* = .33) or BAI (*p* = .61) scores, and the interval between the first and second session, and the interval between the end of the stimulation and the beginning of the task, was equal for both groups (see Table 1).

**Table 1:** Demographic, clinical, and behavioural characteristics

	Sham (N = *17)*	Verum (N = *16)*	p value
*Demographics*			
Age (years)	57 ± 10 (41–70)	55 ± 9 (39–75)	.61^a^
Gender (% men)	11 (65%)	9 (56%)	.62^b^
Education^e^	6 (3–7)	6 (4–7)	.04^b^
IQ estimation	110 ± 14 (82–130)	98 ± 12 (73–123)	.01^a^
Handedness (right)	16 (94%)	14 (88%)	.60^c^
*Clinical measures*			
MMSE	29 (27–30)	29 (28–30)	.36^d^
BDI	1 (0–4)	2 (0–10)	.33^d^
BAI	1 (0–5)	1 (0–11)	.61^d^
*Stimulation measures*			
Interval session 1–session 2 (days)	14 (7–35)	15 (6–28)	.93^d^
Interval stimulation–task (s)	356 (277–800)	319 (240–488)	.053^d^
*Behavioural measures*			
Session 1: RT correct repeat trials (ms)	820 ± 201 (503–1,182)	849 ± 212 (540–1,269)	
Session 2: RT correct repeat trials (ms)	738 ± 210 (483–1,167)	803 ± 191 (479–1,150)	
Session 1: RT correct switch trials (ms)	901 ± 210 (519–1,286)	927 ± 230 (623–1,354)	
Session 2: RT correct switch trials (ms)	842 ± 240 (541–1,286)	901 ± 244 (517–1,324)	
Session 1: Switch costs (ms)	68 (−24–341)	61 (−23–269)	
Session 2: Switch costs (ms)	107 (−5–201)	79 (1–234)	
Session 1: Failed repeat trials (% of total)	.76 (0–4.08)	.73 (0–4.21)	
Session 2: Failed repeat trials (% of total)	.71 (0–4.78)	.55 (0–3.51)	
Session 1: Failed switch trials (% of total)	.37 (0–3.79)	.18 (0–2.19)	
Session 2: Failed switch trials (% of total)	0 (0–1.76)	.74 (0–3.51)	

Values are presented as mean ± standard deviation or median (range) unless indicated otherwise.

*MMSE* mini-mental state examination, *BDI* Beck depression inventory, *BAI* Beck anxiety inventory.

^a^Independent samples t test.

^b^Pearson’s χ^2^ test.

^c^Fisher’s exact test.

^d^Independent samples Mann–Whitney *U* test.

^e^Education level was measured in 7 levels ranging from 1 (no finished education) to 7 (university training).

### Behavioral results

**Figure 1:**
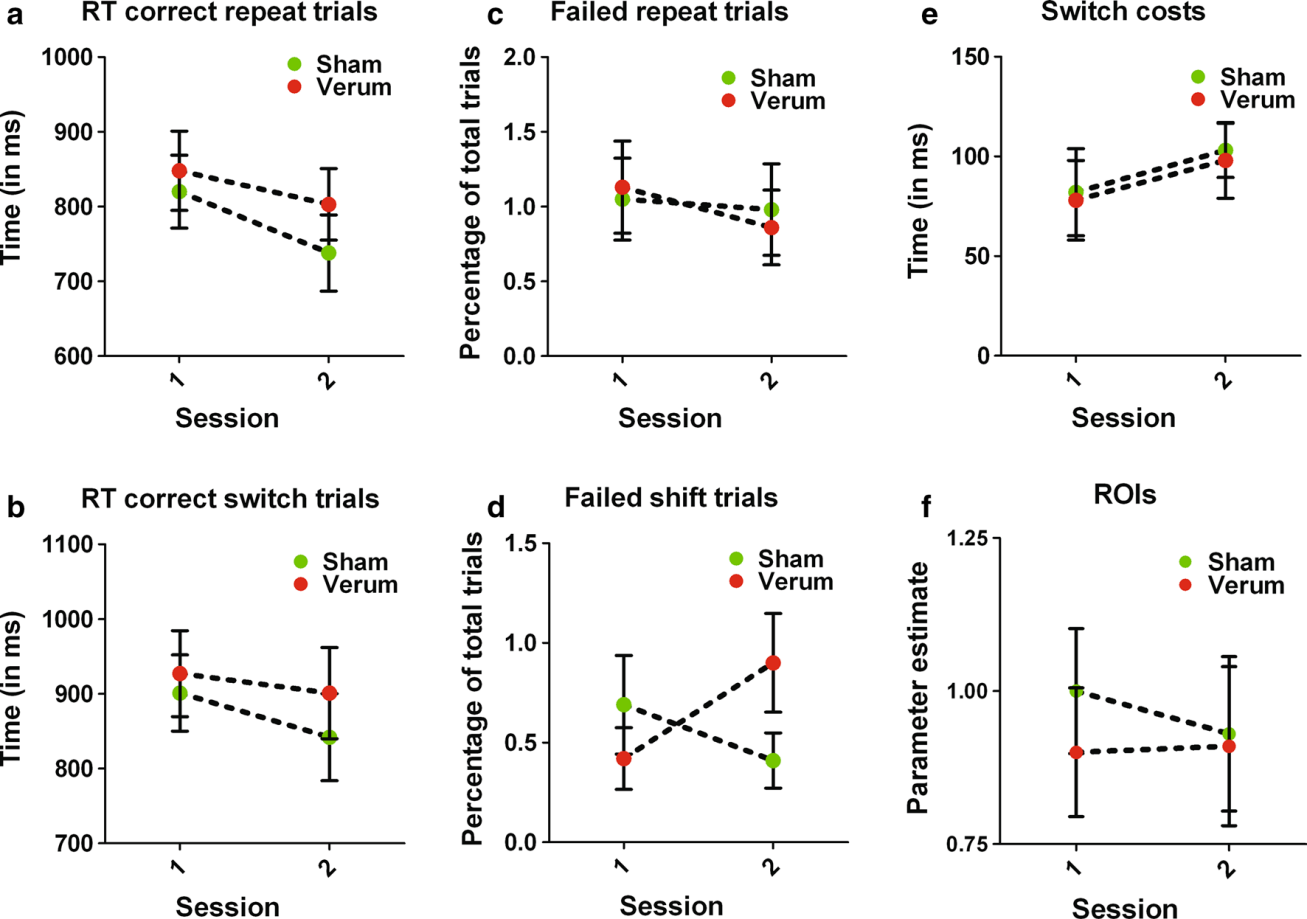
Behavioral data and extracted parameter estimates of the ROIs per group, per session. None of the differences reached significance, except a group-by-session interaction for the failed shift trials (*p* = .04). Post-hoc tests revealed that the verum group made marginally more errors on the second session (*p* = .09). All values represent the mean; *error bars* represent the standard error of the mean (SEM).

The average RTs on correct repeat trials did not differ between the sham and verum group [*F*(1, 31) = 0.47; *p* = .50], but the RTs did decrease from session one to session two [*F*(1, 31) = 12.85; *p* = .001]. This effect was equal for both groups [*F*(1, 31) = 0.92; *p* = .34] (see Figure 1a).

The average RTs on correct switch trials was similar for both groups [*F*(1, 31) = 0.31; *p* = .58]. There was a trend-significant decrease from session one to session two [*F*(1, 31) = 3.02; *p* = .09]. This effect was similar for both groups [*F*(1, 31) = 0.46; *p* = .50] (see Figure 1b).

For the percentage of failed repeat trials, we found no main effect for group [*F*(1, 31) = <.01; *p* = .96], session [*F*(1, 31) = 2.47; *p* = .13], or a group-by-session interaction [*F*(1, 31) = 0.90; *p* = .96] (see Figure 1c).

For the percentage of failed switch trials, we found no main effect for group [*F*(1, 31) = 0.26; *p* = .62] or session [*F*(1, 31) = 0.33; *p* = .57]. There was a significant group-by-session interaction effect [*F*(1, 31) = 4.62; *p* = .04]; post hoc tests indicated that the verum group, compared with the sham group, had a trend-significantly increased percentage of failed switch trials on session two (*U* = 183; *p* = .09), but not on session one (*U* = 112; *p* = .40) (see Figure 1d).

No main effect of group [*F*(1, 31) = 0.04; *p* = .84], session [*F*(1, 31) = 1.52; *p* = .23], or a group-by-session interaction [*F*(1, 31) = <.01; *p* = .98] was found for switch costs (see Figure 1e).

### Imaging results

#### Main effect of task

**Figure 2:**
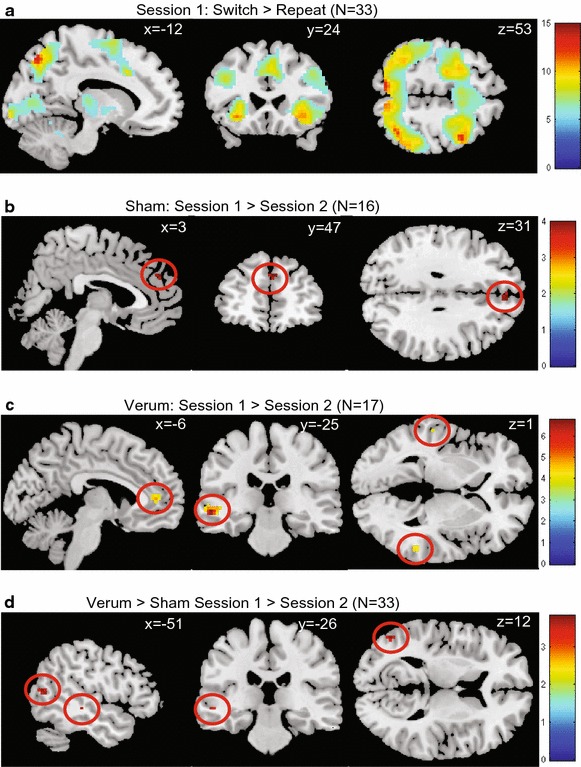
Main effect of task on activity and interaction effects. T-statistic images for the switch > repeat contrast. Threshold at *p* = .05, whole-brain family-wise error-corrected, with an extent of *k* > 10 for the main effect (**a**), and *p* = .001 (uncorrected) with an extent-threshold of *k* > 5 for the interaction effects (**b**, **c**, **d**), overlain on ch2better MNI template with MRIcron. (http://www.mccauslandcenter.sc.edu/mricro/mricron). Coordinates are in MNI space. The *colored bar* indicates the Z values.

We found a robust effect of task (“shift > repeat” contrast) on the first session in the bilateral inferior parietal cortex, left precuneus, bilateral middle frontal gyrus, right middle temporal gyrus, and left inferior temporal gyrus (see Table 2; Figure 2a for the task effects during session one for the whole group; for the task effects per group during session two see Table 3).

**Table 2:** Main effect of task (=shift > repeat) across all subjects on the first session

Area	BA	L/R	t value	Cluster size	Peak coordinates (MNI)		
					X	Y	Z
Inferior parietal cortex	40	L	14.93	3,883	−51	−49	43
	40	R	11.65		48	−46	46
Precuneus	7	L	13.15		−12	−70	49
Middle frontal gyrus	6	R	12.23	5,675	42	5	52
	9	L	11.94		−48	8	37
	46	R	11.29		36	47	25
Middle temporal gyrus	21	R	10.32	2,160	66	−31	−5
	21	R	9.72		57	−25	−11
	37	L	9.93		−51	−61	−8

Shift > Repeat significant at a threshold of *p* = .05 (FWE-corrected) with an extent threshold of *k* > 10.

*BA* Brodmann area.

**Table 3:** Main effects of task (contrast: shift > repeat) on the second session, per group

Area	BA	L/R	t value	Cluster size	Peak coordinates (MNI)		
					X	Y	Z
*Session 2: Sham (N* = *17)*							
Inferior parietal lobe	7	L	16.97	1,401	−33	−52	46
	7	L	14.10		−12	−70	49
	40	R	13.69		36	−52	49
Middle frontal gyrus	46	L	12.91	78	−39	50	19
	46	R	8.87	69	42	44	25
	46	R	8.60		36	50	10
	9	L	9.74	77	−48	8	34
	9	L	9.47		−42	−4	34
	8	L	8.69	91	−27	5	52
	6	L	8.60		−21	−10	52
	6	L	7.30		−39	2	58
	6	R	7.60	23	39	2	55
	6	R	7.04		30	2	64
	6	R	7.04		30	−4	58
	9	R	8.14	39	51	11	43
Inferior frontal gyrus	46	R	7.36		54	20	28
Medial frontal gyrus	6	R	7.38	17	3	14	52
Frontal operculum	47	R	7.93	23	33	23	4
	47	L	8.61	97	−27	20	4
	47	L	7.73		−24	29	−5
Putamen		L	7.43		−21	17	−5
		L	7.56	14	−15	−4	−2
		R	7.13	13	21	14	−5
		R	6.94		21	14	4
Dorsomedial thalamus		R	8.42	20	6	−16	13
		L	9.28	40	−6	−22	10
Inferior temporal gyrus	37	R	7.69	34	48	−55	−14
	37	R	7.25		48	−54	−14
	37	L	9.69	60	−48	−49	−8
	37	L	8.20		−51	−58	−11
	37	L	7.69		−51	−37	−8
Middle occipital gyrus	19	R	6.92		51	−70	−8
	19	L	7.95	13	−36	−79	−17
Cerebellum		R	10.31	146	39	−55	−26
		R	9.21		30	−70	−23
*Session 2: Verum (N* = *16)*							
Inferior parietal cortex	40	L	11.34	266	−51	−40	37
	40	L	10.72		−36	−46	37
	40	L	10.12		−39	−52	46
	40	R	10.40	160	39	−49	40
	40	R	10.26		48	−46	43
	40	R	9.19		54	−37	43
	7	R	10.17	34	9	−73	46
Superior parietal cortex	7	L	8.10	11	−9	−64	52
	7	R	11.05	66	33	−64	49
Superior occipital gyrus	19	R	10.84		33	−73	34
Precentral gyrus	6	L	9.83	98	−30	2	43
Middle frontal gyrus	6	L	9.81		−18	−1	61
	6	L	8.95		−24	−7	58
Middle frontal gyrus	6	R	9.60	48	24	2	58
	6	R	8.67		27	−7	58
	46	L	9.79	24	−45	29	31
Inferior frontal gyrus	45	R	10.44	34	54	23	25
	45	R	8.09		57	11	25
Middle temporal gyrus	39	L	9.62	10	−30	−70	25
Cerebellum		R	13.90	163	30	−64	−20
		R	8.47		36	−52	−32
		L	9.21	122	−27	−64	−26
		L	9.01		−33	−67	−20
		L	8.54		−42	−64	−29

Significant at a threshold of *p* = .05 (FWE-corrected) with an extent threshold of *k* > 10.

*BA* Brodmann area.

#### Group × session interaction effects

During session one, no differences in task-related activation between the two groups were found. Within-group comparisons showed that during the second, compared with the first session, the sham group showed decreased activation of the right medial PFC (see Figure 2b), and the verum group decreased activation of the bilateral temporal cortex and left anterior cingulate cortex (see Figure 2c). For both groups, no areas were more active on the second session when compared with the first. The group-by-session interaction analysis showed that the verum group, when compared with the sham group, activated the left middle temporal cortex more on session one when compared with session two (see Figure 2d). In contrast, the sham group, when compared with the verum group, did not activate more brain areas on the first session when compared with the second session (see Table 2).

Our ROI-based results showed that the activation of the left dorsal PFC did not differ in activity between groups [*F*(1, 31) = .15; *p* = .71] or between sessions [*F*(1, 31) = .17; *p* = .68], and no group-by-session interaction effect [*F*(1, 31) = .26; *p* = .61] were found (see Figure 1f) (Table 4).

**Table 4:** Session and group-by-session interaction effects for the contrast shift > repeat

Area	BA	L/R	t value	Cluster size	Peak coordinates (MNI)		
					X	Y	Z
*Session1: Sham* *>* *Verum*							
No significant results to display							
*Session 1: Verum* *>* *Sham*							
No significant results to display							
*Sham: Session 1* *>* *Session 2*							
Middle frontal gyrus	9	R	3.98	6	3	47	31
*Sham: Session 2* *>* *Session 1*							
No significant results to display							
*Verum: Session 1* *>* *Session 2*							
Middle temporal gyrus	21	L	6.76	44	−57	−22	−8
	21	R	4.46	5	57	−4	−17
	21	R	4.22	6	54	−43	1
	39	L	4.17	28	−48	−64	10
Cingulate gyrus	32	L	5.06	20	−3	44	7
*Verum: Session 2* *>* *Session 1*							
No significant results to display							
*Sham* *>* *Verum Session 1* *>* *Session 2*							
No significant results to display							
*Verum* *>* *Sham Session 1* *>* *Session 2*							
Middle temporal gyrus	22	L	3.83	20	−39	−61	7
	22	L	3.81		−48	−64	10
	21	L	3.57	6	−57	−22	−5

Effects are depicted at *p* = .001 (uncorrected) threshold with an extent threshold of *k* > 5.

#### Connectivity analyses

**Figure 3:**
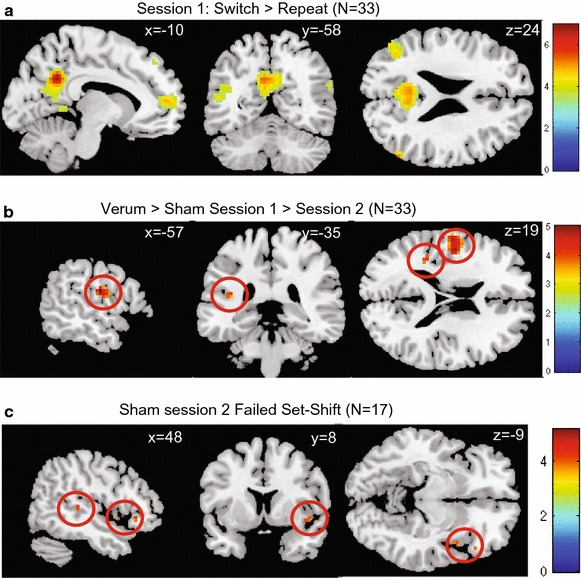
Main effect of task on functional connectivity, interaction effects, and negative correlation between functional connectivity and number of errors on set-shift trials during the second session for the sham group. T statistic images for the switch > repeat contrast. Threshold at *p* = .001 (*uncorrected*) with an extent of *k* > 10 for the main effect (**a**), and *p* = .001 (*uncorrected*) with an extent-threshold of *k* > 5 for the interaction effects and regression (**b**, **c**) overlain on ch2better MNI template with MRIcron (http://www.mccauslandcenter.sc.edu/mricro/mricron). Coordinates are in MNI space. The *colored bar* indicates the Z values.

In both groups, the seed region (the left dorsal PFC) displayed more functional connectivity with the bilateral precuneus, bilateral medial PFC, bilateral inferior parietal cortex, and left superior frontal gyrus during set-shift trials when compared with repeat trials (see Figure 3a; Table 5).

**Table 5:** Main effects of functional connectivity (contrast: shift > repeat) across all subjects per session

Area	BA	L/R	t value	Cluster size	Peak coordinates (MNI)		
					X	Y	Z
*Session 1 (N* = *33)*							
Precuneus	7	L	6.99	423	−6	−52	31
		L	3.87		−9	−46	1
Superior frontal gyrus	8	L	5.40	77	−18	26	52
Medial frontal gyrus	10	R	5.05	201	9	62	7
Anterior cingulate gyrus	32	L	4.84		−6	53	7
	32	R	3.86		6	47	4
Superior temporal gyrus	39	R	5.00	13	57	−61	25
	39	L	4.72	179	−42	−64	28
Angular gyrus	39	L	4.70		−45	−70	34
Middle temporal gyrus	39	L	4.44		−51	−67	16
*Session 2 (N* = *33)*							
Posterior cingulate gyrus	31	L	4.68	205	−12	−55	28
	31	R	4.13		12	−55	25
	31	L	3.75	27	−3	−25	34
Inferior frontal gyrus	45	L	4.42	50	−45	26	19
	46	L	3.69		−45	35	10
	44	L	4.05	22	−51	14	31
Thalamus		R	4.20	10	6	−4	4
Superior temporal gyrus	39	R	3.94	21	48	−55	22
	39	R	3.92		57	−61	16
Middle temporal gyrus	39	L	3.71	11	−51	−61	16

Shift > Repeat significant at a threshold of *p* = .001 (uncorrected) with an extent threshold of *k* > 10.

*BA* Brodmann area.

No between-session differences in functional connectivity were found for either the verum or the sham group during the first session.

The connectivity analyses showed a group-by-session interaction effect: in the verum group, when compared with the sham group, the functional connectivity of the seed regions with the left postcentral gyrus, and left posterior insula was decreased during session two when compared with session one (see Figure 3b; Table 6).

**Table 6:** Session and group-by-session interaction effects in task-related functional connectivity for the contrast shift > repeat

Area	BA	L/R	t value	Cluster size	Peak coordinates (MNI)		
					X	Y	Z
*Session 1: Sham* *>* *Verum*							
No significant results to display							
*Session 1: Verum* *>* *Sham*							
No significant results to display							
*Sham: Session 1* *>* *Session 2*							
No significant results to display							
*Sham: Session 2* *>* *Session 1*							
No significant results to display							
*Verum: Session 1* *>* *Session 2*							
No significant results to display							
*Verum: Session 2* *>* *Session 1*							
No significant results to display							
*Sham* *>* *Verum, Session 1* *>* *Session 2*							
No significant results to display							
*Verum* *>* *Sham, Session 1* *>* *Session 2*							
Precentral gyrus	43	L	5.02	59	−57	−10	22
	30	L	4.75		−48	−7	19
Insula	13	L	4.01	9	−39	−34	19

Effects are depicted at *p* = .001 (uncorrected) threshold with an extent threshold of *k* > 5.

For the sham group, we found that more task-related functional connectivity of the left PFC with the right superior temporal gyrus, right inferior frontal gyrus and right insula correlated negatively with errors on the shift trials. For the verum group, no voxels reached the statistical threshold for this analysis (see also Figure 3c; Table 7).

**Table 7:** Whole brain negative correlations between task-related functional connectivity of the left dorsal prefrontal cortex and the number of errors on set-shift trials per group after rTMS stimulation

Area	BA	L/R	t value	Cluster size	Peak coordinates (MNI)		
					X	Y	Z
*Session 2: Verum*							
No significant results to display							
*Session 2: Sham*							
Middle temporal gyrus	21	R	5.14	30	66	−37	1
Inferior frontal gyrus	47	R	4.27	6	48	26	−5
Superior temporal gyrus	41	R	4.14	7	51	−31	7
Insular cortex	13	R	4.02	6	45	8	−5

Effects are depicted at *p* = .001 (uncorrected) threshold with an extent threshold of *k* > 5.

## Discussion

We investigated the effect of low-frequency rTMS on the left dorsal PFC on behavioural performance, task-related neural activity, and network connectivity in a group of elderly healthy controls, while performing a feedback-based set-shifting paradigm. We found a significant group-by-session interaction effect on errors during set-shift trials after low-frequency stimulation, which was accompanied by a decrease in task-related activation of the left temporal cortex, and a decrease in functional connectivity of the left dorsal PFC with the left postcentral gyrus and posterior insula.

We found the behavioral group-by-session interaction effect only for the percentage of errors on set-shift trials, indicating that the induced ‘virtual lesion’ was selective for this trial type and did not influence performance on repeat trials. Although post hoc tests did not reach significance, the verum group decreased numerically, whereas the sham group increased in performance over sessions, as hypothesised.

Both groups had decreased RTs on correct repeat and correct set-shift trials during session two compared with session one, which we attribute to a learning effect.

Switch costs showed no session, group, or group-by-session interaction effects. Although this measure is an often employed indication for behavioural performance in the set-shifting literature, they are typically a more sensitive measure for performance in rule-based than in feedback-based paradigms [2]. In rule-based paradigms, an extrinsic signal (e.g. background colour) at the beginning of a trial indicates that the presented target stimulus has to be classified according to another feature (i.e. a set-shift trial), thereby capturing the actual mental set-shift, which is typically reflected in increased switch costs. However, because information about an upcoming set-shift makes it less difficult to perform the set-shift, which is reflected in reduced switch costs and errors [23], and because this preparation effect increases with longer preparation time [24, 25], the signalling of an upcoming set-shift trial typically results in an underestimation of the true switch costs, such as in our feedback-based paradigm. The absence of an effect of TMS on switch costs in our study might thus be a true negative finding, or might be due to the insensitivity of this measure in feedback-based set-shifting tasks.

We found robust task-related activation of fronto-parietal areas during the set-shift trials, across both groups, especially in the bilateral parietal cortex, bilateral prefrontal cortex, and bilateral middle temporal gyri. These areas are in accordance with meta-analyses on set-shifting [26–28]. It is important to emphasize that no differences in task-related brain activation between both groups were found during the first session, thus indicating that there were no group differences at baseline.

We found, only for the verum group, decreased activation in the left temporal cortex at the second session, when compared with the first, indicating that inhibiting rTMS decreased the neural activity in task-related brain areas in the left hemisphere. The temporal lobe is associated with context dependent set-shifting [28], a sub-type of set-shifting on which our task also critically depends. We hypothesize that the decrease in activation in the left inferior temporal lobe underlies the increase in errors on the set-shift trials. In contrast to our hypothesis, no modulation of task-related activation was found in the fronto-striatal or fronto-parietal network. We know from previous studies that the effects of rTMS are not limited to the stimulated area, but also affect activity in the circuit that the area is part of, through structural or functional connectivity [11, 14, 29].

Task-related functional connectivity analyses showed that in both groups the left dorsal PFC was more connected with the bilateral precuneus, bilateral medial PFC, bilateral inferior parietal cortex, and left superior frontal gyrus during switch trials when compared with repeat trials. Although some of these areas are associated with the default mode network (DMN) [30], a brain network that becomes active during rest [31] or during a low-demanding baseline condition [32], other theories state that these areas are so-called “flexible hubs” that are essential for relaying information during task performance [33].

We found, only for the verum group, a decrease in functional connectivity of the left dorsal PFC with the postcentral gyrus and posterior insula after rTMS, but not within the a priori expected areas, such as regions within the fronto-striatal or fronto-parietal circuits. Altered task-related functional connectivity between the left dorsal PFC and other task-related brain regions might result in less efficient information processing through the task-related network and consequently results in reduced behavioral performance. The results from the regression analysis on the relation between behavioural task performance and task-related functional connectivity after real versus sham rTMS suggest that higher connectivity between the PFC and right fronto-temporal areas was important for better task performance, and that this relation was absent in the verum group, possibly because of the rTMS. This finding further substantiates the idea that rTMS disturbs healthy brain function and influences behaviour accordingly.

Combining rTMS and task-related fMRI provides insight into both behavioral and neural effects of the experimental modulation on brain excitability. Although we found a behavioral and neural deficit after inhibiting rTMS, the effects were subtle and outside the expected fronto-striatal or fronto-parietal networks. Based on the low percentage of failed set-shift trials in both groups during both sessions, we hypothesize that these networks might not have been optimally challenged due to the simplicity of our task and that therefore the modulation of cortical excitability within these areas might have occurred sub-threshold. We, therefore, recommend future studies to employ a cognitively more demanding behavioral task to investigate the induced neural modulation effects more extensively.

Our findings confirm the importance of the dorsal PFC in executive functioning and corroborate previous findings about how PFC dysfunction can lead to executive dysfunction in diseases such as obsessive–compulsive disorder [3], schizophrenia [4], and Parkinson’s disease [5, 6]. One might extrapolate that using excitatory, instead of inhibitory rTMS, on the PFC could normalize prefrontal functioning, thus leading to improvements in executive functions in these patients, and might potentially provide an adjuvant therapy for cognitive rehabilitation. This hypothesis is further strengthened by a recent meta-analysis that shows that off-line high-frequency brain stimulation on the left DLPFC increases behavioral performance on a working memory task [34].

The current study has some methodological strengths, such as the simplicity of the set-shifting paradigm, the individually fMRI-determined stimulation locations, and investigating the effect of rTMS on both neural task-related neural activity and connectivity. However, using a new developed paradigm also limits the comparability to earlier studies, and, as discussed previously, the paradigm might cognitively not have been demanding enough. From a more methodological perspective, we might have overestimated the stimulation threshold by visually assessing the resting motor threshold when compared with procedures employing EMG. Our rTMS procedure, furthermore, assumes equal cortical excitability in both motor cortex as prefrontal areas. Although this is a commonly employed routine in the TMS literature, it is not necessarily a valid assumption [35]. These two issues should be considered and might be a complication for replication studies in the future. Also, our decision to use optimally targeted, individual fMRI-guided neuronavigation meant that we were bound to stimulate at a second session, possibly leading to learning effects, that potentially could have reduced the effect of our manipulation. Last is the use of off-line rTMS: since the effect of rTMS wears off with time, the time window to measure the effect of the stimulation is only limited. This might be overcome by using on-line stimulation using MRI-compatible TMS equipment.

## Conclusions

To conclude, we applied off-line inhibitory rTMS on the left dorsal PFC in a group of healthy controls while performing a simplified set-shifting paradigm with high construct validity. We found that the participants in the verum group had decreased task-related activity in the left temporal lobe, and displayed reduced functional connectivity of the left dorsal PFC with other task-related areas in the left hemisphere, which was accompanied by a subtle behavioral effect. These results emphasize the importance of the dorsal PFC for adequate executive functions and, furthermore, put forward the possibility of using rTMS in the future as a tool to improve impaired executive functioning in patients with frontal-striatal disorders using excitatory rTMS.

## Methods

### Participants

This cohort of healthy participants was initially recruited as control group for a different study [36], for which we employed the following inclusion criteria: healthy control participants should (1) not suffer from a neurological or psychiatric illness, or have a history of substance abuse, (2) not display cognitive complaints/deficits, (3) not have a history of epilepsy, and (4) match the demographics (i.e. age, sex, education, handedness) of our cohort of Parkinson’s disease patients. Forty healthy participants were randomly appointed to the verum (N = 20) or sham (N = 20) rTMS condition. A number of subjects was, however, excluded from the final analyses due to (1) problems during data acquisition (1 verum), (2) excessive movements (more than 3 mm/3°) during scanning (2 sham; 1 verum), (3) discrepancy between stimulation location and stimulated location (1 verum), and (4) extreme scores on inaccuracy (more than two standard deviations from the median) in comparison with their own group (1 sham; 1 verum), rendering our total sample size 16 participants (mean age of 55 ± 9 years) in the verum rTMS condition and 17 age and gender matched participants (mean age of 57 ± 10 years) in the sham rTMS condition.

We screened all participants for the presence of psychiatric disorders using the Structured Clinical Interview for DSM-IV Axis-I Disorders (SCID-I) [37], depressive symptoms using the Beck Depression Inventory (BDI) [38], anxiety symptoms using the Beck Anxiety Index (BAI) [39], and general cognitive status using the Mini-Mental State Examination (MMSE) [40]. Handedness was assessed using the Edinburgh handedness inventory [41]. The study protocol was reviewed and approved by Research Ethics Committee of the VU University Medical Center (VUmc) and all participants provided informed consent.

### Experimental procedure

Participants were enrolled into a two-arm, randomised and single-blind study design in which they visited the VUmc on three separate occasions. On the first, they performed cognitive tests, were screened for mental disorders, and practiced the set-shifting task. On the second occasion, they performed the set-shifting task in an MRI scanner, after which the fMRI data were analyzed (see below for details). To optimize coil localization [42], we used the fMRI data from the first scanning session to individually determine the coordinates of either the vertex (sham) or the task-related peak-voxel of the “switch > repeat” contrast within the left dorsal PFC (verum). This coordinate was then projected onto the individually acquired T1-weighted scan and used on the third occasion (with an interval of no more than 4 weeks between the second and third occasion) by applying the ASA4.1 neuro-navigation software (ANT Neuro, The Netherlands) to stimulate the individually determined location.

### Stimulation procedure

First, we localized the hand area of the left primary motor cortex using a hand-held figure-of-eight TMS coil (Medtronic MagOption), gradually decreasing the intensity of the individual pulses until a muscle twitch in the right hand was only visually detectable in 5 out of 10 trials. Since longer stimulation at a higher threshold is associated with increased efficacy [43, 44], all participants received 20 min of rTMS at 1 Hz, 110% of the individual motor threshold (1,200 pulses per participant) at either the left dorsal PFC (verum condition) or the vertex (sham condition) in a room adjacent to the MRI scanner. The median time interval between the end of the stimulation and the beginning of the set-shifting task in the MRI scanner was 5:56 min for the sham and 5:19 min for the verum group (no significant difference).

### Set-shifting task

In our in-house developed set-shift task, programmed in E-Prime (version 2.0) and available on request to the authors, an arrow appeared for maximally 4,000 ms either on the left or on the right side of a fixation cross in the centre of the screen, pointing in a downward or an upward direction, leading to four possible response types. The participant had to respond to the stimulus feature (location/direction) that was relevant at the moment of presentation. For instance, if the arrow pointed downwards on the left side of the fixation cross, while location was the correct classification rule, the accurate response was left. A coloured (green = correct; red = incorrect) feedback screen with a fixed duration of 2,000 ms immediately followed a button press. The relevant stimulus feature did not change for four to seven trials (to prevent anticipation) until a red screen followed a correct response, signalling a set-shift. This procedure continued until 40 correct set-shift trials were acquired. The inter-stimulus interval (ISI) between the feedback and stimulus presentation was jittered between 250 and 1,000 ms for anti-aliasing purposes. All behavioural responses were recorded using an MRI compatible response-box (Cambridge Research Systems Ltd., UK).

**Figure 4:**
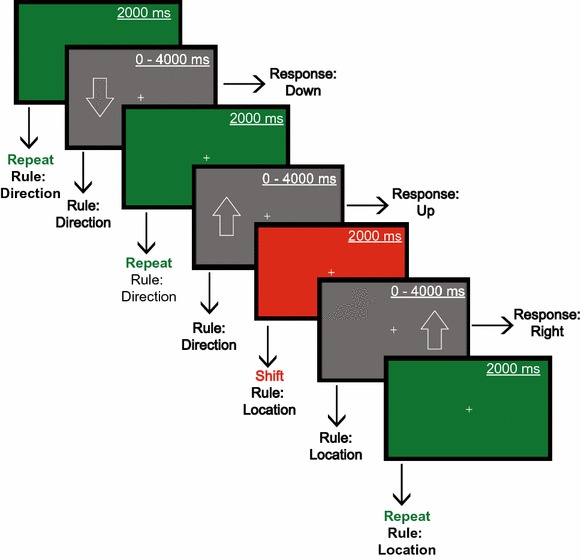
The set-shift paradigm. Stimuli consisted of *arrows* that could appear in two different locations (*left/right*) and point in two different directions (*up*/*down*). The stimulus was presented on the screen for a maximum of 4,000 ms and was terminated upon a button press. Each response was immediately followed by a *green* (correct response) or *red* feedback (incorrect response) screen for 2,000 ms. The correct response depended on the relevant feature of the stimulus (i.e. location/direction). A *red* feedback screen following a correct response signaled a rule shift.

Depending on the version of the task, the arrow was either located above or below the fixation cross (version 1), or on the left and right side (version 2), and it pointed in a left/right or upward/downward direction, respectively. The order of the versions a participant would receive during the first and second session was counterbalanced between both groups. This was done to minimise learning/carry-over effects. Last, we acquainted the participant with the paradigm by practising it extensively prior to the actual recording to exclude learning effects (Figure 4).

### Image acquisition

Imaging was performed at the VUmc using a GE Signa HDxt 3-T MRI scanner (General Electric, Milwaukee). Whole-brain functional images were acquired with a gradient echo-planar imaging (EPI) sequence (TR = 2,100 ms; TE = 30 ms; 64 × 64 matrix; field of view = 24 cm; flip angle = 80°) with 40 ascending slices per volume (3.75 × 3.75 mm in-plane resolution; slice thickness = 2.8 mm; inter-slice gap = 0.2 mm). Structural scanning included a sagittal three-dimensional gradient-echo T1-weighted sequence (256 × 256 matrix; voxel size = 1 × 0.977 × 0.977 mm; 172 sections).

#### Behavioral data

Each behavioural response was classified into the categories (1) “correct repeat” if no set-shift was necessary and the stimulus was correctly categorized, (2) “successful shift” if the preceding feedback signaled a set-shift, and the response was correct, (3) “failed shift” if the preceding feedback signaled a set-shift, but it was not performed, (4) “delayed shift” if a correct shift followed a “failed shift”, (5) “failed repeat” if the participant shifted to the other classification rule without a set-shift signal, (6) “no shift/no repeat” when shifting back to the correct classification rule after a “failed repeat”.

We computed the percentage of failed shift trials per session per participant by dividing the absolute number of failed shift trials by the absolute total number of trials, and multiplying it by 100. A similar procedure was applied for the failed repeat trials. Both measures were used to assess accuracy. We calculated switch costs (=mean reaction time (RT) successful shift − mean RT successful repeat) to assess the cognitive effort to perform a set-shift [2], although we did not a priori expect the groups to differ on this measure since switch costs are most sensitive to rule-based and not feedback-based paradigms. The individually determined average behavioural scores were used in a mixed-model repeated measures design with session (session one/session two) as within-subject factor and group (sham/verum) as between-subject factor in SPSS 20 (SPSS, Chicago, IL, USA). Post-hoc independent samples *t* tests were used to compare the test scores between the groups, and the Mann–Whitney *U* test in case of non-parametric distribution.

#### Image processing and analysis

In SPM8, the EPI scans were first slice-time corrected, realigned to the first image, and unwarped using a least squares approach and a six parameter (rigid body) spatial transformation to correct for motion. They were then warped to the Montreal Neurological Institute (MNI) T1-template, employing the individual T1-weighted image for estimation. Lastly, the images were smoothed with an eight mm Gaussian kernel.

Our subsequently constructed first level general linear model (GLM) event design matrix consisted of two regressors of interest, (1) “successful repeat” (consisting of correct repeat trials) and, (2) “successful shift” (consisting of correct set-shift trials). Both regressors were modelled at the moment of feedback with a fixed duration of 2,000 ms. All other trials, and the six movement parameters that were generated during the realignment were included as regressors of no-interest. Our contrast of interest was “successful shift > successful repeat” (“shift > repeat”). In addition, we computed first level models to assess between session differences per participant. These models were a combination of the first level models of session one and session two and contained the same regressors as previously described. The contrast of interest was “session one > session two, shift > repeat”.

Contrast images derived from the first level analyses were used at second level to investigate (1) within-group, between-session differences employing paired *t* tests (2) between group differences per session employing independent *t* tests and (3) group differences in between session differences employing independent *t* tests. Brain regions were identified using the WFU-Pick Atlas [45]. Whole-brain statistical maps were thresholded at *p* < .05 corrected for family-wise errors (FWE) in the main effects with an extent-threshold of *k* > 10, and at *p* < .001 uncorrected, with a voxel extent-threshold of *k* > 5, after masking inclusively for the main effects for the group interaction effects to be sensitive to small, yet meaningful differences.

#### Regions of interest

**Figure 5:**
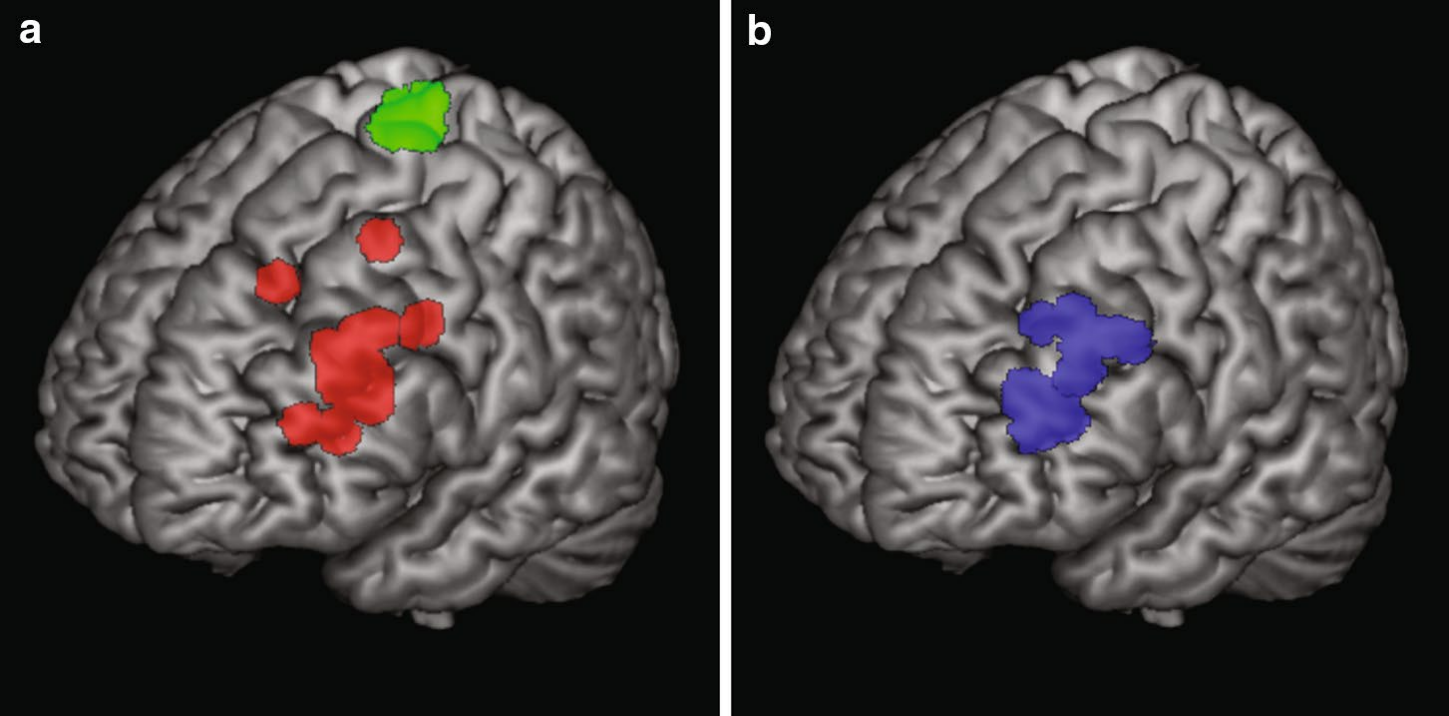
Illustration of all stimulation locations/regions-of-interest. **a** Summary illustration of the individual stimulation locations for the sham (=*green color*) and the verum group (=*red color*). **b** Summary illustration of the locations of the individual peak-voxels within *left* prefrontal cortex for the sham group (=*blue*). These areas represent the areas that would have been stimulated if the participants had been placed in the experimental group. The *red* and *blue* ROIs were used to extract the average parameter estimates using *MarsBar* and served as seed region for the connectivity analyses. 5 mm spherical ROIs were constructed with *MarsBar*, and are overlaid on ch2better MNI template rendering with MRIcron (http://www.mccauslandcenter.sc.edu/mricro/mricron) for illustrative purposes.

We defined 5 mm spherical regions-of-interest (ROIs) at the rTMS stimulation location for the verum group, and at the location of the peak-voxel in the left dorsal PFC in the sham group that would have been the locus of stimulation if they had been placed in the verum group, using MarsBar (http://marsbar.sourceforge.net) (see Figure 5a, b). Each subject-specific ROI was subsequently masked with the first-level activity mask to exclude task-unrelated voxels. Then, we extracted the average parameter estimates of the whole ROI, using the task effect contrast, per session, per participant. Lastly, we compared the average parameter estimates in a mixed-model repeated measures design with session (session one/session two) as within-subject factor and group (sham/verum) as between-subject factor.

#### Functional connectivity: gPPI

We assessed task-related functional connectivity of the stimulated areas in the verum group and of the selected ROIs in the left PFC in the sham group using a generalized form of context-dependent psychophysiological interaction (gPPI) [46, 47]. A gPPI analysis statistically tests in a whole-brain voxel-wise manner whether areas outside the seed region are functionally connected to the seed region during the task [47]. We used the individually determined ROIs described in the previous paragraph as seed regions.

At first-level, our contrast of interest was “shift > repeat”, now using the PPI terms that were convoluted with the seed region time-course, and leaving the psychological variable (task conditions) and movement parameters as covariates of no interest. All further constructed contrasts-of-interest were identical to the activity-based analyses described previously.

We also performed a whole-brain analysis of the negative relationship (using a regression analysis) between task-related functional connectivity of the left PFC during the second session and the percentage of failed shift trials, per group separately, to assess the potential influence of changes in functional connectivity on behavioural performance.

Whole-brain statistical maps were thresholded at *p* < .001 uncorrected, with a voxel extent-threshold of *k* > 10 for the main effects, and a voxel extent-threshold of *k* > 5 for the interactions and regression. We also masked inclusively for the main effect of group or session for the interaction effects, to be sensitive to subtle, but relevant, task-related effects.

## Abbreviations

rTMS: repetitive transcranial magnetic stimulation (rTMS)
PFC: prefrontal cortex
DLPFC: dorsolateral prefrontal cortex
fMRI: functional magnetic resonance imaging
MMSE: Mini-Mental State Examination
SCID-I: Structured Clinical Interview for DSM-IV Axis-I Disorders
BDI: Beck Depression Inventory
BAI: Beck Anxiety Inventory
DMN: default mode network
ROI: region-of-interest
gPPI: generalized form of context-dependent Psycho-Physiological Interaction
